# A Systematic Strategy for Discovering a Therapeutic Drug for Alzheimer’s Disease and Its Target Molecule

**DOI:** 10.3389/fphar.2017.00340

**Published:** 2017-06-19

**Authors:** Zhiyou Yang, Tomoharu Kuboyama, Chihiro Tohda

**Affiliations:** Division of Neuromedical Science, Department of Bioscience, Institute of Natural Medicine, University of ToyamaToyama, Japan

**Keywords:** Alzheimer’s disease, axonal regeneration, CRMP2 phosphorylation, DARTS analysis, memory recovery, Drynaria rhizome

## Abstract

Natural medicines are attractive sources of leading compounds that can be used as interventions for neurodegenerative disorders. The complexity of their chemical components and undetermined bio-metabolism have greatly hindered both the use of natural medicines and the identification of their active constituents. Here, we report a systematic strategy for evaluating the bioactive candidates in natural medicines used for Alzheimer’s disease (AD). We found that Drynaria Rhizome could enhance memory function and ameliorate AD pathologies in 5XFAD mice. Biochemical analysis led to the identification of the bio-effective metabolites that are transferred to the brain, namely, naringenin and its glucuronides. To explore the mechanism of action, we combined the drug affinity responsive target stability with immunoprecipitation-liquid chromatography/mass spectrometry analysis, identifying the collapsin response mediator protein 2 protein as a target of naringenin. Our study indicates that biochemical analysis coupled with pharmacological methods can be used in the search for new targets for AD intervention.

## Introduction

The development of drugs from natural medicines is a powerful and promising research track. Because of the diversity of constituents in herbal medicines, multi-component interactions and multiple molecular targets must be considered, making it difficult to investigate the signaling mechanism. *In vitro* bioassay-guided isolation is one of the typical ways to access active compounds (Koehn and Carter, 2005) and provides a window into the mechanisms of herbal drugs. However, this approach does not always detect the actual active compounds because this method ignores the bio-metabolism of constituents before and after absorption (Wishart, 2016). The BBB permeability of these metabolites is one of the crucial factors in the development of a medicinal drug for central nervous system (CNS) diseases. In other words, the application of natural medicines to the treatment of neurodegenerative diseases is largely hindered by the limited knowledge of their metabolism, the effective metabolites and their brain transfer, and the molecular mechanisms underlying this metabolism. Therefore, addressing which metabolites are delivered to the brain is at the forefront of research to unravel the true mechanisms of natural medicines.

Liquid chromatography-mass spectrometry (LC-MS) analysis has been a powerful tool for identifying micro constituents from the bio-samples obtained after the oral delivery of herbal drugs (Xiao et al., 2012). Based on LC-TOF/MS analyses, a few reports have shown selected brain-penetrating metabolites to be interventions for AD (Wang et al., 2012; Ho et al., 2013), but their targets and mechanisms were not well clarified. Our laboratory and others have shown that the drug affinity responsive target stability (DARTS) method is effective in identifying the target protein of a ligand (Lomenick et al., 2009; Tohda et al., 2012). Using this method, we explored the signaling of several small molecules (Tohda et al., 2012; Shigyo et al., 2015). In the present study, we established a series of procedures to investigate which proteins bind to the compounds and confirm the interaction using LC-MS and DARTS analyses to identify brain-active compounds after the oral administration of a drug.

Here, we construct a systematic strategy to unravel the mechanisms of action of the natural medicines used for AD treatment by exploring the brain-penetrating metabolites and their target proteins as a starting point. Briefly, the steps include (1) screening of effective natural medicines that may enhance axonal elongation and recover memory function using *in vitro* and *in vivo* models, which have been established previously (Tohda et al., 2004; Kuboyama et al., 2005); (2) systematically exploring effective metabolites delivered to the brain using chemical and pharmacological analyses; (3) identification of the target proteins as an initial point in identifying the signaling axis of effective metabolites; and (4) clarification of the specific signaling pathway driven by those identified metabolites. In this study, using DR as an example, we first found that DR was able to recover memory deficits in a murine AD model. By performing a systematic approach utilizing LTQ Orbitrap Fourier Transform Mass Spectrometry (LTQ-Orbitrap-FT-MS/MS), we identified real active metabolites that were distributed in the brain after the oral administration of DR. Furthermore, the target of the active metabolites was identified by a combination of DARTS and LTQ-Orbitrap-FT-MS/MS. We are the first to identify the effective form of DR and its mechanism for AD treatment.

## Materials and Methods

### Animal Studies

All animal experiments were carried out in accordance with the Guidelines for the Care and Use of Laboratory Animals of the Sugitani Campus of the University of Toyama. All protocols were approved by the Committee for Animal Care and Use of the Sugitani Campus of the University of Toyama. The approval number for the animal experiments is A2014INM-1, and the confirmation number for the recombinant gene experiments is G2013INM-1.

Transgenic mice (5XFAD) were obtained from Jackson Laboratory (Bar Harbor, ME, United States). They were maintained as double hemizygotes by crossing with B6/SJL F1 breeders. To test the effect of the DR extract on 5XFAD mice, we used 5XFAD mice (male and female, 6–8 months old) and non-transgenic wild-type littermate mice (male and female, 6–8 months old). To investigate the effect of naringenin on 5XFAD mice, we used 5XFAD mice (male, 8–12 months old) and non-transgenic wild-type littermate mice (male, 8–12 months old). All mice were housed with free access to food and water and were kept in a controlled environment (25 ± 2°C, 12-h light/dark cycle starting at 7:00 am).

### Behavioral Test

The DR extract was prepared as described previously (Yang et al., 2015). The DR extract was dissolved in saline to concentrations of 0.5, 5, and 50 mg ml^-1^. The DR extract or vehicle solution (saline) was orally administered once per day for 31 days. On day 20 of DR administration, mice were individually habituated to an open-field box composed of polyvinyl chloride (33 cm × 28 cm; height, 26.5 cm) for 10 min. Their paths were tracked using a digital camera system. The distance moved for 10 min was considered the locomotor activity and was analyzed with Etho Vision 3.0 (Noldus, Wageningen, Netherlands). At days 21, 25, and 31, the object recognition test (ORT), object location test (OLT), and episodic-like memory test (ELM) were performed as described previously (Kuboyama et al., 2015). Naringenin (Sigma–Aldrich, St. Louis, MO, United States) was suspended in saline to a concentration of 0.5 or 10 mg ml^-1^ and was orally administered once per day for 31 days. On days 21, 22, and 29 of naringenin administration, the open-field locomotion test, ORT, and OLT were performed.

### Immunohistochemistry

After the behavioral studies, mice were deeply anesthetized with chloral hydrate (Wako, Tokyo, Japan) and perfused with saline followed by ice-cold 4% paraformaldehyde. Dissected brains were post-fixed in 4% paraformaldehyde for 24 h at 4°C, immersed in 30% sucrose for 48 h for cryoprotection, and stored at -30°C. The brains were cut into 20 μm coronal slices (six slices of each mouse brain) every 100 μm in the perirhinal cortex area (bregma -1.46 to -2.06 mm) using a cryostat (Leica, Heidelberg, Germany). The slices were fixed with 4% paraformaldehyde and stained with a polyclonal antibody against Aβ(1–40/42) (1:300) (Chemicon, Temecula, CA, United States, cat # AB5076), a monoclonal antibody against pNF-H (1:300) (Covance, Emeryville, CA, United States, cat # SMI-35R), and a monoclonal antibody against PHF-tau (1:100) (Thermo Scientific, Rockford, IL, United States, cat # MN1020) at 4°C for 24 h. Alexa Fluor 488-conjugated goat anti-rabbit IgG (1:300) and Alexa Fluor 594-conjugated goat anti-mouse antibody (1:300) were used as secondary antibodies (Molecular Probes, Eugene, OR, United States, cat # ab150077 and ab150116, respectively). Fluorescence images of axons, PHF-tau and Aβ(1–40/42) were captured using a fluorescent microscope (BX-61/DP70, Olympus, Tokyo, Japan) at 324 mm × 430 mm. Six successive slices from the perirhinal cortex containing the hippocampus (CA1, CA3, and DG) of each mouse were captured for quantification. The images were analyzed with ImageJ (NIH) as described previously (Tohda et al., 2012).

### Bioavailability, Metabolism, and Brain Penetration of DR Extract

The DR water extract was re-dissolved in H_2_O to a concentration of 10 mg ml^-1^ and filtered with a 0.45 μm membrane; a 10 μl sample was used for LC-MS analysis. To explore the metabolites that penetrated the brain, DR or vehicle solution (saline) was orally administered to 5XFAD mice (male and female, *n* = 3) at a concentration of 13 g kg^-1^ for a single dose. Thirty minutes and 5 h after drug administration, mice were euthanized and blood was collected. The plasma was obtained after centrifugation of blood at 11,000 *g* for 10 min at 4°C. The brain cortex was dissected following perfusion with saline. Plasma (200 μl) was extracted with methanol, dried, and resolubilized in 50 μl methanol. The brain cortex was homogenized and extracted with methanol, dried, and resolubilized in water (containing 0.1% formic acid) before loading onto preactivated Sep-Pak C_18_ SPE cartridges (Waters). The metabolites were eluted with methanol, vacuum dried, sonicated, and resolubilized in mobile phase before LC-MS analysis. To calculate the concentration of metabolites in the brain, the standard curve was made. Simply, the standard compounds were mixed with blank cortex and extracted as the method above, and then were applied to LC-MS analyses. A Thermo Scientific^TM^ Accela HPLC system interfaced with an LTQ Orbitrap XL hybrid Fourier Transform Mass Spectrometer (Thermo Fisher Co., San Jose, CA, United States) was used to chemically profile DR and the biosamples. Liquid chromatographic analysis was performed on a Capcell Pak C_18_ MGIII S-5 (1.5 mm i.d. × 150 mm, Shiseido, Tokyo, Japan) column held at 40°C with a flow rate of 200 μl/min. In the mobile phase, 0.1% aqueous formic acid (v/v) (A) and acetonitrile (B) were used. The following linear elution gradient was used: 0–5 min, 6–20% B; 5–10 min, 20–30% B; 10–25 min, 30–70% B; 25–26 min, 70–6% B; 26–32 min, 6% B. The following ESI parameters were used: spray voltage 4.5 kV, capillary voltage 40.0 kV, tube lens 150 V, capillary temperature 330°C, sheath gas flow rate 50 units, and aux gas flow rate 10 units. We operated the mass spectrometer in the positive ESI mode, scanning from 50 to 2,000 m/z, and calibrated the instrument using a polytyrosine solution before each experiment.

### Primary Cultures and Measurement of Axonal Density

Embryos were removed from a pregnant ddY mouse (SLC, Shizuoka, Japan) at 14 days of gestation as described previously (Tohda et al., 2012). The cells were treated with 10 μM Aβ_25-35_ or 5 μM Aβ_1-42_ (Sigma–Aldrich, A4559 or A9810) for 3 days before being treated with naringenin or vehicle (0.1% DMSO) for 4 days. The Aβ_25-35_ or Aβ_1-42_ was previously incubated at 37°C for 4 or 7 days for aggregation, respectively. The cells were fixed with 4% paraformaldehyde and immunostained at 4°C for 24 h with a monoclonal antibody against pNF-H (1:500) as an axonal marker and a polyclonal antibody against MAP2 (1:2000, Abcam, Cambridge, United Kingdom, cat # ab32454) as a neuronal marker. Alexa Fluor 488-conjugated goat anti-rabbit IgG (1:200) and Alexa Fluor 594-conjugated goat anti-mouse IgG (1:200) were used as secondary antibodies. Fluorescence images were captured using a fluorescence microscope system (BX61/DP70, Olympus) at 644 μm × 855 μm. The lengths of the pNF-H-positive axons were measured using a MetaMorph analyser (Molecular Devices, Sunnyvale, CA, United States).

### DARTS Analysis

A mouse brain cortex was homogenized with M-PER (Thermo Scientific) containing 1× protease inhibitor cocktail (Thermo Scientific). The brain lysate (2.7 mg each) was added to 60 μl of 6 mg ml^-1^ DR extract or vehicle solution and incubated for 60 min at room temperature. The mixture was proteolysed using 2.7 mg thermolysin (Sigma) in reaction buffer [50 mM Tris–HCl (pH 8.0), 50 mM NaCl, 10 mM CaCl_2_] for 30 min at 37°C. To stop the proteolysis, 0.5 M EDTA (pH 8.0) was added to each sample in a 1:10 ratio. The samples (600 μg each) were then analyzed using 2D-PAGE. The separated proteins were stained using the colloidal Coomassie method. The spots that were thicker in the DR-treated lysate than the vehicle-treated lysate were cut out and prepared for mass spectrometry analysis. Protein spots were excised, digested with trypsin (Promega, Madison, WI, United States), mixed with α-cyano-4-hydroxycinnamic acid in 50% acetonitrile/0.1% TFA, and subjected to MALDI-TOF analysis (Microflex LRF 20, Bruker Daltonics). DARTS and western blot analysis were performed to confirm the collapsin response mediator protein 2 (CRMP2) protein as a target of naringenin. A primary culture of mouse cortical neurons (ddY, E14) was maintained for 72 h without drug treatment. The cells were lysed, and cell lysate (15 μg each) was added to 1.67 ml of 10 mM naringenin in vehicle solution and incubated for 60 min at room temperature. The mixture was proteolysed using 3.75 ng thermolysin in reaction buffer for 15 min at 37°C. The reaction was stopped by adding 0.5 M EDTA (pH 8.0) to each sample at a 1:10 ratio. The samples were then subjected to SDS–PAGE for western blot analysis. Anti-CRMP2 (1:3000) (Sigma, St. Louis, MO, United States, cat # C2993) and anti-β-actin (1:1000) (Cell Signaling Technology, cat # 4967) antibodies were used as the primary antibodies, and HRP-conjugated goat anti-rabbit IgG (1:2000) (Santa Cruz, CA, United States, cat # sc-2004) was used as the secondary antibody.

### IP Coupled with LC-MS Analysis

Mouse primary cortical neurons (E14) were cultured for 3 days before 100 μM naringenin was added for 0.5, 2, or 6 h. After being washed three times with 1× PBS, neuronal cells were lysed with M-PER solution. Thirty micrograms of protein from each sample was denatured and extracted with 100 μl methanol, and the supernatant was subjected to LC-MS after centrifugation at 14,000 *g* for 10 min. For immunoprecipitation (IP), the lysates (30 μg) treated with naringenin for 6 h were incubated with antibody (CRMP2 or IgG, 3.5 μg) for 1 h before being incubated with protein G dynabeads (Life Technologies) for 10 min at 4°C. The beads were washed three times with PBS containing 0.02% Tween 20 and then extracted with methanol. The supernatant was applied to LC-MS after centrifugation for 10 min at 14,000 *g*. The LC-MS conditions were the same as those in the aforementioned experiment except for the linear elution gradient: 0–5 min, 20–50% B; 5–20 min, 50–90% B; 20–21 min, 90–20% B; 21–27 min, 20% B.

### siRNA Knockdown

siRNAs were transfected into mouse cortical neurons (ddY E14) together with GFP vectors according to the manufacturer’s protocol for nucleofection (Lonza, Basel, Switzerland). Briefly, mouse cortical neurons (5.0 × 10^6^ cells) were mixed with siCRMP2 (1 μM, Thermo Scientific, cat # 4390771) or control siRNA (1 μM, Thermo Scientific, 4390843) and GFP vector (2 μg, Lonza, Basel, Switzerland) and electroporated with an Amaxa Nucleofector (Lonza). Three days after the transfection, cells were fixed and immunostained with a polyclonal antibody against CRMP2 (Sigma, cat # C2993). Double immunostained neurons were selected for statistical analysis. The appropriate concentration of siRNA for knockdown was determined previously. Three days after the transfection of siRNA, the cells were treated with naringenin (10 μM) or vehicle solution (0.1% DMSO). Four days after treatment, the axon density was measured.

### *In Vitro* Evaluation of Phosphorylated CRMP2

Mouse primary cortical neurons (E14) were cultured for 3 days and then treated with 10 μM Aβ_25-35_ for another 3 days. After removal of the Aβ-containing medium, the cells were treated with naringenin (1 μM) for 0.5, 24, or 96 h. Cells were fixed and double immunostained with a polyclonal antibody against pThr514 CRMP2 (1:200) (Cell Signaling Technology, cat # 9397) and a monoclonal antibody against MAP2a/2b (1:200, Fremont, CA, United States, cat # MS-249-S) as a neuron marker. The expression of pCRMP2 was measured using a CS Analyzer (ATTO, Tokyo, Japan).

### Statistical Analysis

Graphs were generated with GraphPad Prism 5 (GraphPad Software, La Jolla, CA, United States), and Student’s unpaired *t*-test, one-way analysis of variance (ANOVA) with Dunnett’s *post hoc* test and two-way ANOVA with Bonferroni *post hoc* test were performed for statistical analysis of the data, and the statistical significance criterion *P*-value was 0.05. The data are presented as the mean ± SEM.

## Results

### DR Extract Ameliorates Memory Deficits in the 5XFAD Mouse Model

**FIGURE 1:**
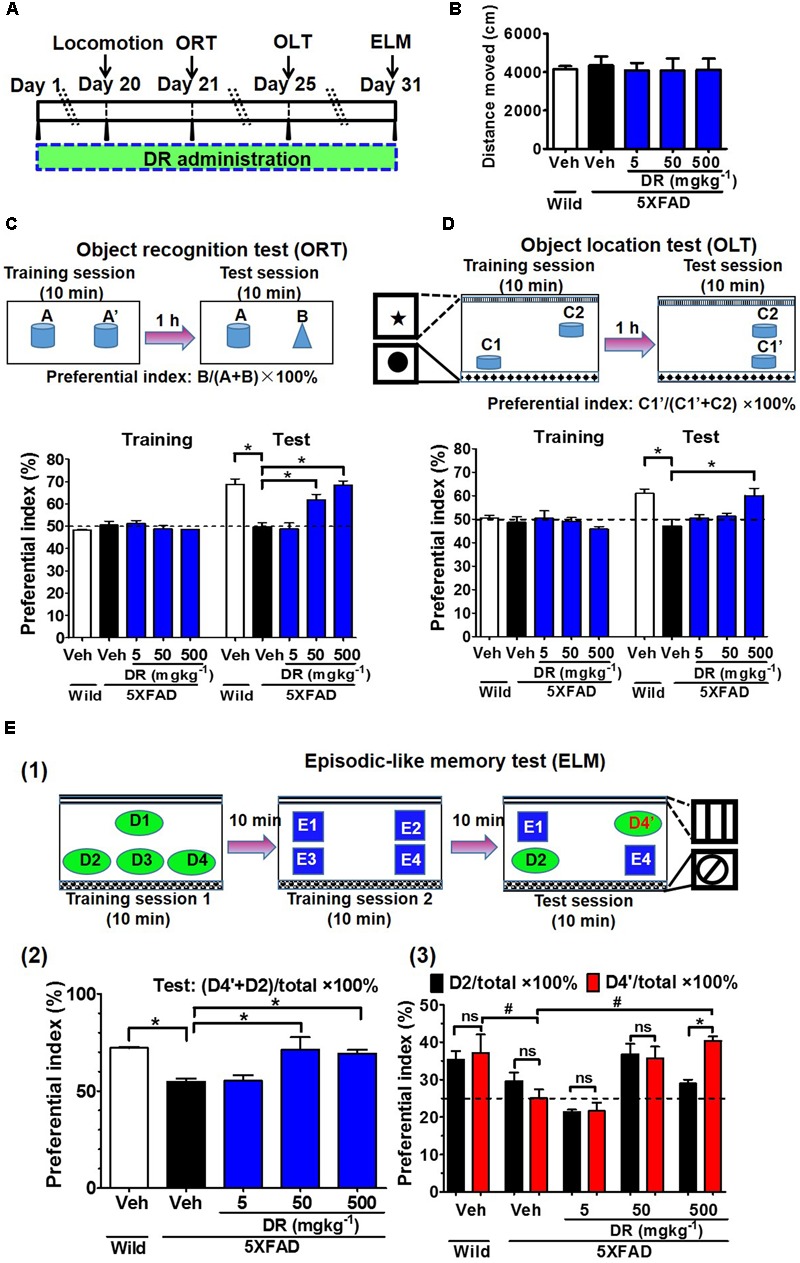
DR extract rescues memory deficits in 5XFAD mice. **(A)** Time course of the experiments. **(B–E)** Effects of DR extract on the open-field test, object recognition memory, object location memory, and episodic-like memory. Six- to eight-month-old wild-type mice and 5XFAD mice were used (*n* = 3–5). **(B)** Total distance moved (within 10 min) in the open-field test. **(C)** Object recognition test. The preference indices for objects A’ (training session) and B (test session) were quantified. *p* < 0.0001, drug × time interaction was analyzed using repeated measures two-way ANOVA, *F*(4,15) = 13.18. ^∗^*p* < 0.05, *post hoc* Bonferroni test. **(D)** Object location test. The preference indices for objects C1 (training session) and C1’ (test session) were quantified. *p* = 0.007, drug × time interaction was analyzed using repeated measures two-way ANOVA, *F*(4,15) = 5.34. ^∗^*p* < 0.05, *post hoc* Bonferroni test. **(E)** Episodic-like memory test. **(E1)** Time schedule of ELM. **(E2)** The preference indices for objects D4’ plus D2 (test session) were quantified. ^∗^*p* < 0.05, one-way ANOVA *post hoc* Bonferroni test. **(E3)** The preference indices for objects D2 (black columns) and D4’ (red columns) were also quantified. ^#^*p* < 0.05, two-way ANOVA *post hoc* Bonferroni test. ^∗^*p* < 0.05, one-way ANOVA *post hoc* Bonferroni test.

DR extracts (5, 50, or 500 mg kg^-1^ per day) or vehicle solutions were orally administered to 5XFAD mice. The determination of DR doses was according to the animal study of other groups (Anuja et al., 2010; Lee et al., 2014). The memory test scheme was showed in **Figure 1A**. In the object recognition memory test (**Figure 1C**), all of the mice showed equivalent exploratory behavior toward each of the objects (preference indices were approximately 50%) in the training session. In the test session, wild-type mice and DR (50 and 500 mg kg^-1^ per day)-treated 5XFAD mice exhibited significantly more frequent exploratory behavior toward a novel object than vehicle-treated 5XFAD mice (approximately 50%). Improvement of recognition memory by DR (500 mg kg^-1^ per day) was confirmed by other independent experiments, too (Supplementary Figures 1A,B).

To test whether spatial memory is improved by DR, we performed the object location memory test (**Figure 1D**); in this test, in which one of the novel objects was located in a different place in the test session, the vehicle-treated 5XFAD mice could not distinguish the object with the novel location from the object with the familiar location, whereas wild-type mice and DR (500 mg kg^-1^ per day)-treated 5XFAD mice significantly increased their exploratory behavior toward the object in the novel location. The ELM was carried out to evaluate the ability of the mice to integrate object (what), location (where), and context recognition (when) (Dere et al., 2005). In this test, among the four objects in the test session, E1 and E4 were located at the same places in training session 2, while the objects D2 and D4’ had been presented to mice in training session 1 (**Figure 1E(1)**). Therefore, objects D2 and D4’ were more unfamiliar than objects E1 and E4 in the test session. Wild-type mice and DR (50 and 500 mg kg^-1^ per day)-treated 5XFAD mice significantly increased their exploratory behavior toward the unfamiliar objects (D2 and D4’) compared with the familiar objects (E1 and E4) (**Figure 1E(2)**). The vehicle-treated 5XFAD mice failed to distinguish between the presented objects. For objects D2 and D4’, D2 was presented to mice at the same location in training session 1. D4’ was located at a novel place for the mice; therefore, if the mouse memorized object places and presentation orders, the mouse would show higher preference indices for object D4’ than D2. Like the vehicle-treated 5XFAD mice, the wild-type mice failed to recognize objects D2 and D4’; in contrast, DR (500 mg kg^-1^ per day)-treated 5XFAD mice significantly increased their exploratory behavior toward object D4’ rather than D2 (**Figure 1E(3)**), this might due to the memory improvement of DR on normal mice (Supplementary Figures 2A–C). The animals’ body weights were recorded during the drug administration, with no significant changes observed in any of the groups (Supplementary Figure 2D). In the open-field test, no significant locomotor differences were detected in the distances moved and velocity among the five groups (**Figure 1B** and Supplementary Figure 2E).

Collectively, these results indicate that DR extract rescues cognitive deficits when orally administered to 5XFAD transgenic mice.

### DR Extract Ameliorates AD-Like Pathology in 5XFAD Mice

**FIGURE 2:**
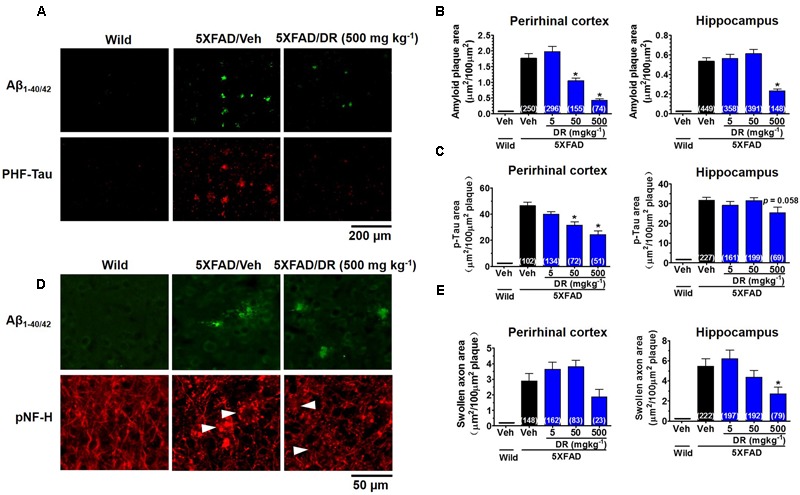
DR extract ameliorates AD-like pathology in 5XFAD mice. DR extract (5, 50, 500 mg kg^-1^, p.o.) or vehicle solution (saline) was administered to wild-type or 5XFAD mice (males and females, 6–8 months old) for 31 days. The day after the behavioral test, mice were sacrificed and subjected to brain analysis. **(A)** Representative images of Aβ_1-40/42_-positive plaques and PHF-tau in the perirhinal cortex. **(B)** The total area of amyloid plaques per 100 μm^2^ was quantified in the cerebral cortex and hippocampus. **(C)** The total area of PHF-tau associated with Aβ plaques per 100 μm^2^ was quantified in the cerebral cortex and hippocampus. **(D)** Aβ plaques and axons were double immunostained with Aβ_1-40/42_ and pNF-H antibodies. pNF-H-positive bulb- or ring-like axonal structures were localized with Aβ plaques. Representative images from the perirhinal cortex are shown. **(E)** The total area of abnormal axons per 100 μm^2^ of amyloid plaque in the cerebral cortex and hippocampus are shown. One-way ANOVA and Dunnett’s *post hoc* test were used for all statistical analyses (^∗^*p* < 0.05 vs. veh/5XFAD; the number of measured areas is shown in each column).

All of the aforementioned wild-type mice and 5XFAD mice were euthanized after the behavior tests. To examine the effect of orally administered DR on the number of Aβ plaques, the brains of mice were sectioned and stained for Aβ_1-42_ to visualize Aβ plaques. We measured Aβ plaques in the perirhinal cortex and hippocampus of the brain. Amyloid deposits were primarily localized in the cerebral cortex and hippocampus of 5XFAD mice, while no Aβ deposits were observed in the age-matched wild-type mice (**Figure 2A** and Supplementary Figure 2F). DR treatment dose-dependently reduced Aβ plaques in the cortex of 5XFAD mice and diminished Aβ plaques in the hippocampus at the 500 mg kg^-1^ per day dose (**Figure 2B**).

To assess the level of paired helical filament (PHF)-tau, another key neuropathological feature in AD, we stained the brain slices with an anti-phosphorylated tau antibody. PHF-tau was observed very close to the Aβ plaques (**Figure 2A**) in 5XFAD mice, while no PHF-tau was observed in the wild-type mice. DR dose-dependently decreased the levels of PHF-tau in the cortex, and a decreasing trend in PHF-tau was observed in the hippocampus after DR treatment (**Figure 2C**).

As DR stimulated axonal outgrowth after Aβ_25-35_-induced atrophy in cultured primary cortical neurons (Yang et al., 2015), we investigated whether any changes were induced by DR treatment in 5XFAD mice. Abnormal axon structures, such as bulb- or ring-like neurofilaments stained with pNF-H, are located within amyloid plaques in both preclinical and end-stage AD patients (Dickson and Vickers, 2001). Our previous study also indicated that bulb-shaped axons are colocalized with amyloid plaques in 5XFAD mice (Tohda et al., 2012). The axons in the vehicle-treated 5XFAD mice were blocked at the front of amyloid plaques and formed swollen bulb- or ring-like shapes (**Figure 2D**). Abnormal axons were rarely detected in the distal part of amyloid plaques. DR (500 mg kg^-1^ per day) treatment significantly reduced the swollen axonal areas in the brain cortex and hippocampus (**Figure 2E**). Collectively, these results indicate that orally administered DR effectively repairs degenerated axons in the brains of 5XFAD mice, which might contribute to the recovery of memory function.

### DR-Derived Metabolites Penetrate the BBB after the Oral Administration of DR Extracts to 5XFAD Mice

**FIGURE 3:**
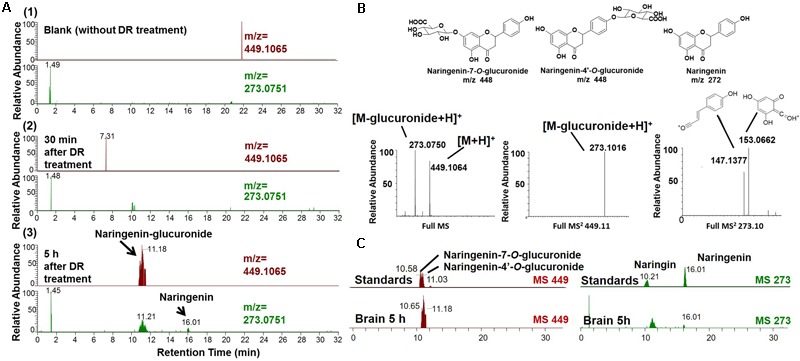
Brain-penetrating metabolites after the oral administration of DR extracts to 5XFAD mice. DR extract (13 g kg^-1^, p.o.) or vehicle solution (saline) was administered to mice (male and females, 12 months old, *n* = 3) as a single dose. Mice were sacrificed 0.5 or 5 h after treatment, and the brain cortex was extracted with methanol and subjected to LC-MS analysis. **(A)** Extracted ion current (EIC) chromatograms of naringenin glucuronide (m/z = 449.1065) and naringenin (m/z = 273.0751). **(A1)** Without DR treatment; **(A2)** 30 min after DR treatment; **(A3)** 5 h after DR treatment. **(B)** Proposed structures of the DR metabolites identified as naringenin-7/4′-*O*-glucuronide and naringenin (Upper) and mass fragmentation characteristics of naringenin glucuronide (Lower). **(C)** Comparison of the EIC chromatograms for naringenin-7/4′-*O*-glucuronide, naringenin, and naringin standards and samples obtained 5 h after DR treatment.

To clarify the mechanism through which DR may function in treating AD, we tried to detect DR-derived metabolites that are delivered into the brain. Using the high-accuracy quasi-molecular ion ([M+H]^+^) and a mass error of ±1 mmu, we comprehensively characterized 17 compounds by comparing their MS-MS data and fragmentation patterns with those of reference standards or reported compounds (Supplementary Figure 3 and Table 1). Naringin and neoeriocitrin are the two main compounds in DR extract, with contents of 96.8 and 108.0 mg g^-1^, respectively. Naringenin was present at 0.27 mg g^-1^ in DR extract. Naringenin and naringenin glucuronide are metabolized from naringin and were detected in the plasma of rats after the oral administration of naringin (Fang et al., 2006). Then, we profiled the chemicals that were delivered into the blood and brain in 5XFAD mice with HPLC-FT-MS. Usually higher doses than effective doses are selected to detect the metabolites of herbal extracts (Xiang et al., 2011), formulations (Fujiwara et al., 2011; Kushida et al., 2013), and even single compounds (Durairajan et al., 2012; Tan et al., 2013) in biosamples. Therefore, we orally administered a high dose of DR (13 g kg^-1^) to the 5XFAD mice. Using SIEVE software (Niu et al., 2015), we performed differential analyses of the total ion current (TIC) chromatograms for the biosamples from vehicle-treated and DR-treated mice (Supplementary Figure 4). The different TIC peaks were analyzed and considered based on their retention times, accurate mass values, and MS-MS fragment patterns. Thirteen compounds or metabolites were detected in the plasma of DR-treated 5XFAD mice, and 3 chemicals were detected in the brain cortex of DR-treated 5XFAD mice (Supplementary Table 1). The extracted ion current (EIC) chromatograms of the speculated brain-delivered metabolites are shown in **Figure 3A**. These metabolites were detected in the brains 5 h after administration of DR (**Figure 3A(3)**) but not detected in the brains of vehicle-treated 5XFAD mice (**Figure 3A(1)**) or 30 min after the oral administration of DR extract (**Figure 3A(2)**). To confirm the structures of the speculated metabolites, we synthesized naringenin-7-*O*-glucuronide and naringenin-4′-*O*-glucuronide from naringenin and analyzed the mass fragmentation characteristics of these compounds (**Figure 3B**). The HPLC-MS results showed similar retention time and MS-MS fragmentations in the standards and DR-treated mouse brain samples (**Figure 3C**). Our pre-experiment showed that the *C*_max_ of metabolites was 5 h after DR administration. Using the standard curve, the calculated levels of naringenin, naringenin-7-*O*-glucuronide, and naringenin-4′-*O*-glucuronide metabolite in the brain are 15.1 ± 2.9, 302.6 ± 77.9, and 22.7 ± 17.8 pmol g^-1^ following DR treatment, respectively. Taken together, these results suggest that the metabolites naringenin and glucuronides of naringenin at the 4′ or 7 position can penetrate the BBB after the oral administration of DR extract.

### DR-Derived Brain Penetrating Metabolites Enhance Axonal Growth in Cultured Neurons after Aβ Treatment

**FIGURE 4:**
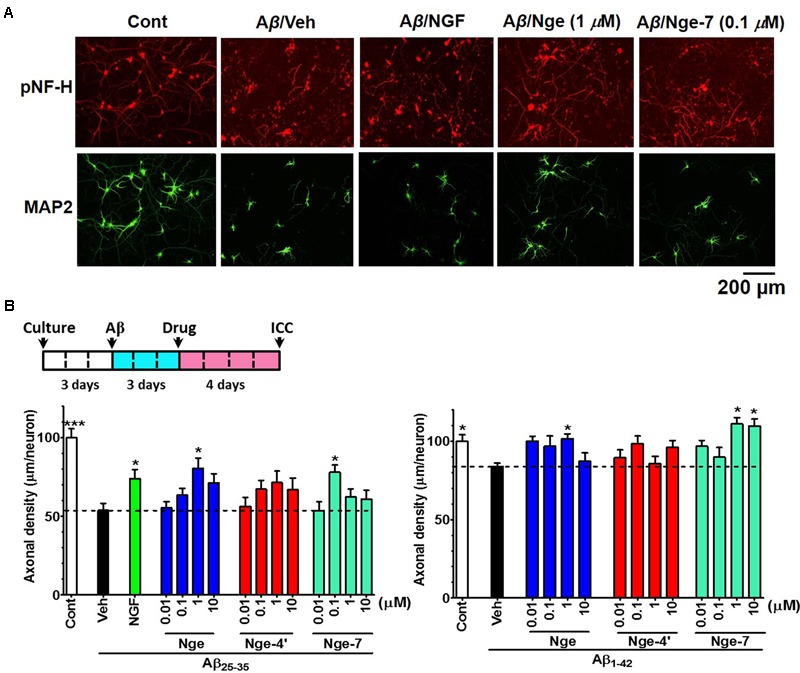
Effects of the metabolites naringenin-7/4′-*O*-glucuronide (Nge-7 or Nge-4′) and naringenin (Nge) on axonal regrowth after Aβ-induced axonal atrophy. Primary cortical neurons were cultured for 3 days, and then the cells were treated with 10 μM Aβ_25-35_ or 5 μM Aβ_1-42_ (Veh). Three days later, the Aβ was removed and one of the compounds (0.01, 0.1, 1, and 10 μM), NGF (100 ng/ml), or vehicle (0.1% DMSO) was added to the neurons. Four days after the treatment, the neurons were fixed and immunostained for pNF-H and MAP2. The lengths of the pNF-H positive neurites were measured. **(A)** Representative images of pNF-H-positive axons and MAP2-positive neurons in Aβ_25-35_ treated group. Scale bar, 200 μm. **(B)** Time course of the experiments (Upper). Quantification of total axonal outgrowth and associated statistics (Lower). The error bars represent SEM. One-way ANOVA and Dunnett’s *post hoc* test were performed (^∗^*p* < 0.05 vs. Aβ/Veh; *n* = 10–40 photographs).

To test the effects of brain-penetrating metabolites on axonal growth, we investigated their effects on both Aβ_1-42_ and Aβ_25-35_-induced axonal atrophy in cultured cortical neurons. According to the bioavailability of DR (13 g kg^-1^) *in vivo*, we calculated the total accumulated naringenin metabolites in the brain at a concentration of 13.1 nM after DR (500 mg kg^-1^) administration. Therefore, we selected the concentration of metabolites from 10 to 10 μM for the study *in vitro*. Axonal density was decreased significantly by Aβ_25-35_ or Aβ_1-42_ treatment, whereas treatment with naringenin (1 μM) and naringenin-7-*O*-glucuronide (0.1 μM) significantly increased the length of pNF-H (phosphorylated neurofilament H, axonal marker)-positive axons (**Figures 4A,B**). What’s more, naringenin (1 μM) and naringenin-7-*O*-glucuronide (1 and 10 μM) significantly reversed Aβ_1-42_ induced axonal atrophy (**Figure 4B**). NGF was used as a positive control for axonal extension after Aβ_25-35_ treatment (Kuboyama et al., 2005). This result indicates that naringenin and naringenin-7-*O*-glucuronide can restore Aβ-induced axonal atrophy.

### Naringenin Ameliorates Memory Deficits and AD-Like Pathology in 5XFAD Mice

**FIGURE 5:**
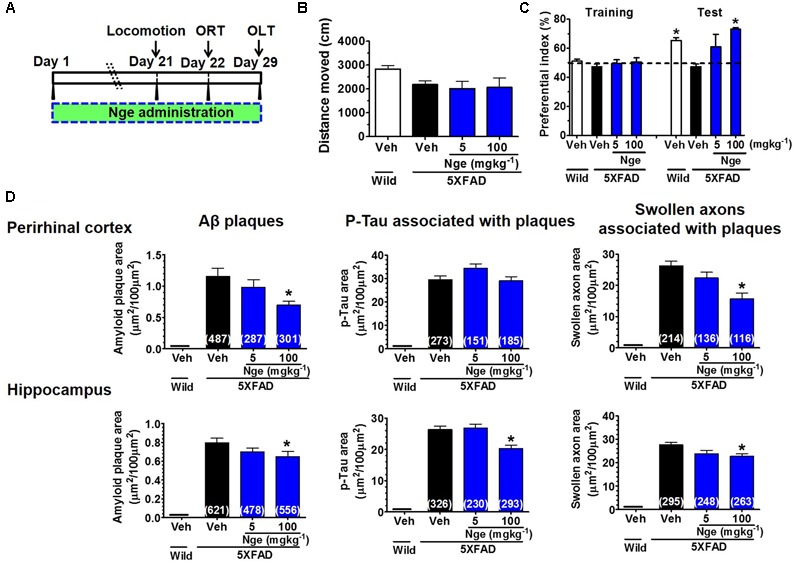
Naringenin ameliorates recognition memory deficits and AD pathologies in 5XFAD mice. **(A)** Time course of behavioral tests. Naringenin (0, 5, or 100 mg kg^-1^ per day) was orally administered to 5XFAD mice (male, 8–12 months old, *n* = 4–5) for 29 days, and their behavioral performance was evaluated. **(B)** Total distance moved (within 10 min) in the open-field test. **(C)** Object recognition test. The preferential indices for the training and test sessions are shown. *p* = 0.009, drug × time interaction was analyzed using repeated measures two-way ANOVA, *F*(3,11) = 6.24. ^∗^*p* < 0.05 vs. vehicle-treated 5XFAD mice, *post hoc* Bonferroni test. **(D)** The total area of amyloid plaques, PHF-tau associated with amyloid plaques, and swollen axons associated with amyloid plaques were measured in the perirhinal cortex and hippocampus. One-way ANOVA and Dunnett’s *post hoc* test were performed in all statistical analyses (^∗^*p* < 0.05 vs. vehicle-treated 5XFAD mice).

According to the contents of naringin and naringenin in DR extract, and naringin could be metabolized to naringenin *in vivo* according to our experiment and other reports. We calculated the total amount of naringenin aglycone in DR extract. Twenty-three milligram naringenin is contained in 500 mg DR extract. We also referred to the dose of naringenin in reports of other AD models (Yang et al., 2014; Ghofrani et al., 2015). We orally administered naringenin (5 and 100 mg kg^-1^ per day) to the 5XFAD mice (male, 8–12 months old) for 31 days (**Figure 5A**). In the ORT (**Figure 5C**), wild-type mice and naringenin 100 mg kg^-1^ per day-treated 5XFAD mice exhibited significantly more frequent exploratory behavior toward the novel object than vehicle-treated 5XFAD mice. In the OLT, naringenin 100 mg kg^-1^ per day-treated 5XFAD mice showed the trend of increased object location memory (Supplementary Figure 5B). There were no significant differences in the distances moved by the four groups in the open-field test (**Figure 5B**). During drug administration, the animals’ body weights did not change significantly (Supplementary Figure 5A).

To examine the effect of orally administered naringenin on AD-like pathology in 5XFAD mice, we performed immunohistochemistry using antibodies against Aβ_1-42_ and PHF-Tau. As shown in **Figure 5D**, no amyloid plaques, PHF-tau, or degenerated axons were observed in the wild-type mice. The naringenin (100 mg kg^-1^ per day)-treated 5XFAD mice exhibited a significant reduction in amyloid plaques and swollen axon expressions (associated with the plaques) in the perirhinal cortex and hippocampus. The PHF-tau expression in the hippocampus (associated with the plaques) was significantly decreased by naringenin (100 mg kg^-1^ per day) treatment. Taken together, the findings revealed that orally administered naringenin ameliorates memory deficits and effectively decreases amyloid plaques, plaque-associated PHF-tau and swollen axons in 5XFAD mice, as shown for DR administration.

### Naringenin Targets CRMP2 and Reduces the Phosphorylation of CRMP2 in Cultured Primary Neurons

**FIGURE 6:**
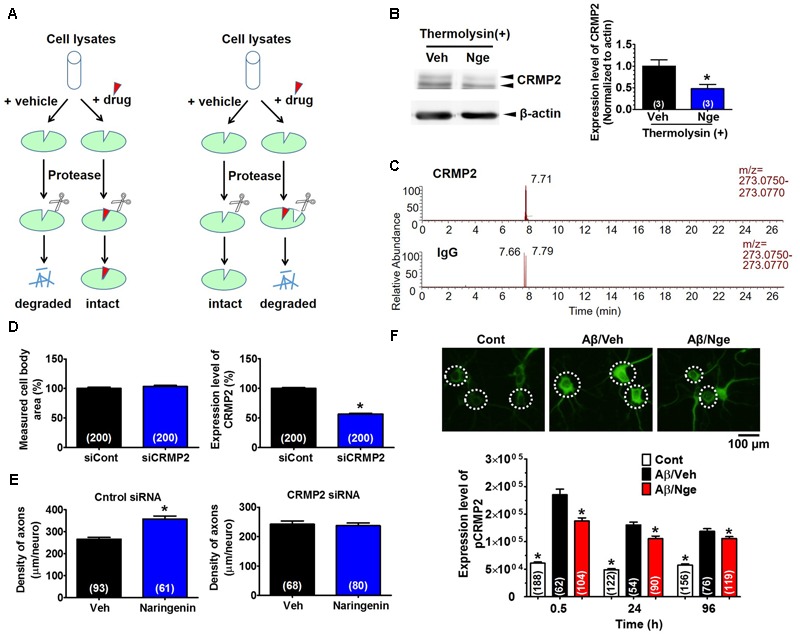
CRMP2 is a target protein of naringenin and is involved in naringenin-induced axonal regrowth. **(A)** Illustration of the DARTS method. **(B)** Identification of CRMP2 as a target of naringenin by DARTS and western blot. Mouse cortical neurons were lysed, naringenin or vehicle solution was incubated with lysates for 1 h at room temperature. The mixture was proteolysed using thermolysin and electrophoresed for western blot [Student’s unpaired *t*-test, ^∗^*p* < 0.05 vs. Veh (T+). The number of repeated experiments is shown in each column], full-length blots are presented in Supplementary Figure 9. **(C)** Confirmation of the binding between naringenin and CRMP2 by IP coupled with LC-MS analysis. Primary cultured neurons were maintained for 3 days and then treated with naringenin for 6 h. Cell lysates were immunoprecipitated with a CRMP2 (Upper) or normal IgG (Lower) antibody, and the pellets were extracted with methanol and subjected to LC-MS. **(D)** Three days after transfection with siCRMP2 (1 μM) and GFP vector (2 μg), the neurons were stained for a CRMP2 antibody. The level of CRMP2 in the GFP-positive neurons was quantified. **(E)** CRMP2 siRNA (1 μM) or control siRNA (1 μM) with GFP vector was transfected into cortical cells. Two days later, the cells were immunostained for pNF-H after treated with 10 μM naringenin or vehicle (0.1% DMSO) for 4 days. The pNF-H-positive axons in the GFP-positive neurons were quantified. **(F)** Naringenin treatment decreased the Aβ-induced phosphorylation of CRMP2. Cortical neurons were cultured for 3 days, treated with Aβ_25-35_ or vehicle solution (dH_2_O) for another 3 days, and then treated with naringenin (1 μM) or vehicle (0.1% DMSO) for 0.5 (representative photos), 24, or 96 h after the removal of the Aβ-containing medium. The cells were double immunostained for pCRMP2 and MAP2. The level of pCRMP2 in neurons was quantified. **(D–F)** The number of neurons is shown in each column. Student’s unpaired *t*-tests were performed in **(D,E)** (^∗^*p* < 0.05), and one-way ANOVA with Dunnett’s *post hoc* test was performed in **(F)** (^∗^*p* < 0.05 vs. Aβ/Veh).

To explore the mechanism responsible for naringenin-induced axonal growth and memory improvement, we first used the DARTS method (Lomenick et al., 2009; Pai et al., 2015) to identify a target protein of naringenin. When a compound binds to a target protein, the protein conformation is probably changed, changing the protease sensitivity of the target protein (Lomenick et al., 2009). Based on our previous study, a 62-kDa protein spot was thicker in the DR-treated brain lysate than the vehicle on a 2D-PAGE gel after thermolysin reaction (Supplementary Figure 6), MALDI-TOF-MS analysis indicated that the spot was possibly CRMP2. To confirm CRMP2 as a target protein of naringenin, we performed DARTS and western blot analyses using cell lysates from mouse primary cultured cortical neurons (**Figure 6A**). After thermolysin treatment, the CRMP2 level in naringenin-treated lysates was significantly decreased compared with the vehicle solution-treated group (**Figure 6B** and Supplementary Figure 9).

Next, to confirm the binding of naringenin to CRMP2, we performed LC-MS combined with IP analysis. First, we evaluated whether naringenin could be taken up by the neurons. We found that naringenin accumulated in the neurons in a time-dependent manner (Supplementary Figures 7A,B). Surprisingly, naringenin glucuronides were also detected in the neurons 30 min after incubation (Supplementary Figure 7A). Then, lysates were prepared from neurons after 6 h of incubation with naringenin and were subjected to IP with a CRMP2 antibody or normal IgG antibody. Naringenin was detected abundantly after IP with the CRMP2 antibody (peak area: 169,940) but was rarely detected in the IgG immunoprecipitated sample (peak area: 8,265) (**Figure 6C**). The single peak detected in the group without the CRMP2 antibody might be caused by a small amount of non-specific binding between protein G and the CRMP2 protein (Supplementary Figure 7C).

To investigate whether CRMP2 mediates naringenin-induced axonal extension, CRMP2 was knocked down using siRNA transfection. Three days after the transfection of siRNA targeting CRMP2 (1 μM), the CRMP2 level in the GFP-transfected neurons was significantly reduced (44%) compared with the control siRNA-transfected neurons (**Figure 6D**, Right). There were no changes in the measured cell body area (**Figure 6D**, Left). In control siRNA-transfected neurons, the axon density was significantly increased by naringenin treatment (**Figure 6E**). However, the naringenin-induced axonal growth was completely abolished by transfection of CRMP2 siRNA (**Figure 6E**). These data indicate that CRMP2 is a target of naringenin and is essential for naringenin-induced axonal growth.

CRMP2 is critical for the regulation of neurite outgrowth due to its binding to tubulin heterodimers (Fukata et al., 2002). Hyperphosphorylated CRMP2 is involved in the Aβ-induced impairment of cognitive memory and is characteristic of Alzheimer’s disease (Gu et al., 2000; Cole et al., 2007; Soutar et al., 2009; Isono et al., 2013). CRMP2 is phosphorylated by Cdk5 at Ser522 and subsequently phosphorylated by GSK-3β at Ser518, Thr514, and Thr509 (Uchida et al., 2005). Therefore, we investigated the effect of naringenin on the expression of pThr514-CRMP2 in Aβ-treated primary cultured cortical neurons. Compared with the control group, CRMP2 was hyperphosphorylated at Thr514 after Aβ_25-35_ treatment; the pCRMP2 level decreased significantly after naringenin treatment (**Figure 6F**).

Collectively, our findings revealed that naringenin binds to CRMP2 and reduces the Aβ-induced phosphorylation of CRMP2, resulting in axonal growth facilitation.

## Discussion

Generally speaking, it is challenging and time-consuming to isolate, identify, and screen the bioactive components in natural medicines. In addition, the components may be metabolized to phase I and II derivatives following absorption. Therefore, the bioavailability and access of metabolites or natural components to brain tissues are absolutely critical factors in predicting the potential for those compounds as CNS drugs. Thus, studies investigating the bioactivity and physiological mechanisms of natural medicines must use the metabolites that have been identified in the target tissues. Here, we report a new strategy that couples biochemical analysis with pharmacological methods and can be used to evaluate therapeutic candidates for AD in natural medicines. Using this method, we found that a crude drug, DR, ameliorated memory deficits and AD-like pathologies in 5XFAD model mice. To clarify the mechanism of action for DR in the treatment of AD, we first identified metabolites that were delivered into the brain. To detect enough concentration and a variety of metabolites as possible as we can, we administered a single dose of DR (13 g kg^-1^) to the mice. As a result, only 3 metabolites were identified in the brain cortex with comprehensive FT-MS-MS analysis.

Previously, we identified five main compounds in the water extract of DR using bioassay-guided isolation, with two of them (neoeriocitrin and caffeic acid-4-*O*-glucoside) showing significant axonal elongation effects after Aβ_25-35_-induced atrophy (Yang et al., 2015). In the present study, these two compounds were detected in the blood but not in the brain after the oral administration of DR (Supplementary Table 1), demonstrating that they could not penetrate the BBB and enter into the brain. Naringin did not show axonal regrowth activity and was not detected in the brain. Nonetheless, two metabolites of naringin—naringenin and naringenin-7-*O*-glucuronide—were found in the brain after the oral administration of DR, and these metabolites reversed Aβ-induced axonal atrophy. Here, we directly identified the metabolites delivered into the brain after an oral dose of a crude extract. Therefore, this method avoids the identification of false-positive compounds that occurs with standard bioassay-guided isolation methods and may be a fast, economical, and efficient way to identify effective metabolites for use as interventions for AD.

Glucuronidation mediated by UDP-glucuronosyltransferases (UGTs) is an important metabolic pathway that facilitates the clearance of xenobiotics, including drugs and environmental substances, and functions in the metabolism of endogenous compounds (Rowland et al., 2013). These hydrophilic glucuronides are generally inactive and show poor BBB permeability, while some glucuronides, such as morphine-6-glucuronide and quercetin-3-*O*-glucuronide, exist in the brain and show important biological activities (Ho et al., 2013; Klimas and Mikus, 2014). Our present report also shows that naringenin-7-*O*-glucuronide can penetrate the BBB and enhance axonal regrowth. How do these glucuronides exist in the brain? One possible explanation is the existence of a transporter at the BBB. The organic anion transporter (OAT) is localized at the membrane of BBB cells and is responsible for the transportation of numerous endogenous and xenobiotic amphipathic compounds (Kullak-Ublick et al., 2001; Sugiyama et al., 2003; Ohtsuki et al., 2004). Moreover, OAT can actively transport morphine-6-glucuronide across the BBB (Bourasset et al., 2003). However, there are no reports regarding a naringenin-glucuronide transporter. Another explanation is the finding of UGTs in the brain and neurons. In the brain, UGTs are mainly expressed in endothelial cells and astrocytes of the BBB (Ouzzine et al., 2014). The expression of glucuronosyltransferase isoform UGT1A6 was reported in primary cultures of neurons and astrocytes (Suleman et al., 1998). Our results also provide evidence that UGTs are present in neurons (Supplementary Figure 7A). Thus, it is possible that naringenin was absorbed by neurons and glucuronidated by UGTs after penetrating the BBB.

Drug affinity responsive target stability has been used for the identification of ligand targets by identifying the alterations in proteolytic sensitivity that occur after the binding of ligand and receptor (Lomenick et al., 2009). Therefore, this method might be used to identify new targets for the treatment of AD. Here, we first combined FT-MS-MS with the DARTS method to discover a brain-penetrating compound, naringenin, and its target protein CRMP2. Actually, DARTS is based on the conformation changes after drug binding, resulting in a decrease or an increase in proteolytic susceptibility of the target protein (Lomenick et al., 2009). In this study, DR showed increased band density while naringenin showed decreased band density of CRMP2 compared with vehicle treated thermolysin group in DARTS analysis. This might due to non-specifically binding of DR derived components (except for naringenin) with CRMP2 in case of DARTS used DR extract, because the proteolytic susceptibility of CRMP2 might be changed compared with that of CRMP2 induced by naringenin. Since naringenin-7-*O*-glucuronide showed axonal regrowth activity after Aβ-induced axonal atrophy, it is possible that naringenin-7-*O*-glucuronide binds to CRMP2. Thus, we performed the DARTS experiment of naringenin and its glucuronides using lysed neurons. The result was shown in Supplementary Figure 8, except for naringenin, naringenin-7-*O*-glucuronide but not naringenin-4′-*O*-glucuronide also showed the possible binding possibility to CRMP2. This was corresponding to the *in vitro* assay result, which indicated that naringenin-induced cellular signaling was attributed to naringenin and naringenin-7-*O*-glucuronide.

Naringenin has been reported to improve learning and memory in Aβ or streptozotocin-injected AD models (Yang et al., 2014; Ghofrani et al., 2015), which might relate to the mitigation of lipid peroxidation and apoptosis or the increased insulin and insulin receptor expression in the rat brain. Naringenin also showed anti-inflammatory activity in LPS/IFN-γ-stimulated glial cells and neuroprotective effects against H_2_O_2_-induced hyper-oxidation (Heo et al., 2004; Vafeiadou et al., 2009). In the present study, we found that naringenin enhanced axonal growth in both normal neurons and neurons subjected to Aβ-induced axonal atrophy. This growth might contribute to the reconstruction of neural networks and improvements in memory. With the exception of lacosamide, which reduced the association between Cdk5-phosphorylated CRMP2 and CaV2.2 *in vitro* and *in vivo* (Moutal et al., 2016), the binding of small molecules to CRMP2 has rarely been reported. Moreover, lacosamide inhibited CRMP2-dependent tubulin polymerization, which prevents the growth of neurites. The present study is the first to describe a molecule, naringenin that targets CRMP2 and inhibits CRMP2 phosphorylation at Thr514. This might restore the association between CRMP2 and tubulin, leading to axonal regrowth.

In summary, we first reported that DR enhances memory function and ameliorates AD pathologies in 5XFAD mice. LTQ-Orbitrap-FT-MS-MS and DARTS analysis pinpointed the effective substances in DR and identified a molecule that binds to CRMP2. This systematic strategy might contribute to the development of natural medicines and exploration of their mechanisms. Moreover, this strategy might be broadly applicable to the identification of novel compounds and targets for intervention in CNS diseases.

## Author Contributions

ZY, TK, and CT designed the experiments and wrote the manuscript. ZY performed the experiments and analyzed the data. CT supervised all experiments and analyses.

## Conflict of Interest Statement

The authors declare that the research was conducted in the absence of any commercial or financial relationships that could be construed as a potential conflict of interest.
