# Supplementary material for: A Systematic Strategy for Discovering a Therapeutic Drug for Alzheimer’s Disease and Its Target Molecule
Source: Front Pharmacol. 2017 Jun 19;8:340. doi: 10.3389/fphar.2017.00340 (PMC5474478; doi:10.3389/fphar.2017.00340)
Supplement: Supplementary file 1 [file Data_Sheet_1.docx]

**A systematic strategy for discovering a therapeutic drug for Alzheimer’s disease and its target molecule**

Zhiyou Yang^a^, Tomoharu Kuboyama^a^, and Chihiro Tohda^a,^*****

**^a^Division of Neuromedical Science, Department of Bioscience, Institute of Natural Medicine, University of Toyama, 2630 Sugitani, Toyama 930-0194, Japan**

***Correspondence: Chihiro Tohda, Ph.D.**

**2630 Sugitani,**

**Toyama 930-0194, JAPAN**

**Phone & FAX: +81-76-434-7646**

**E-mail : chihiro@inm.u-toyama.ac.jp**

**Supplementary Information**

**
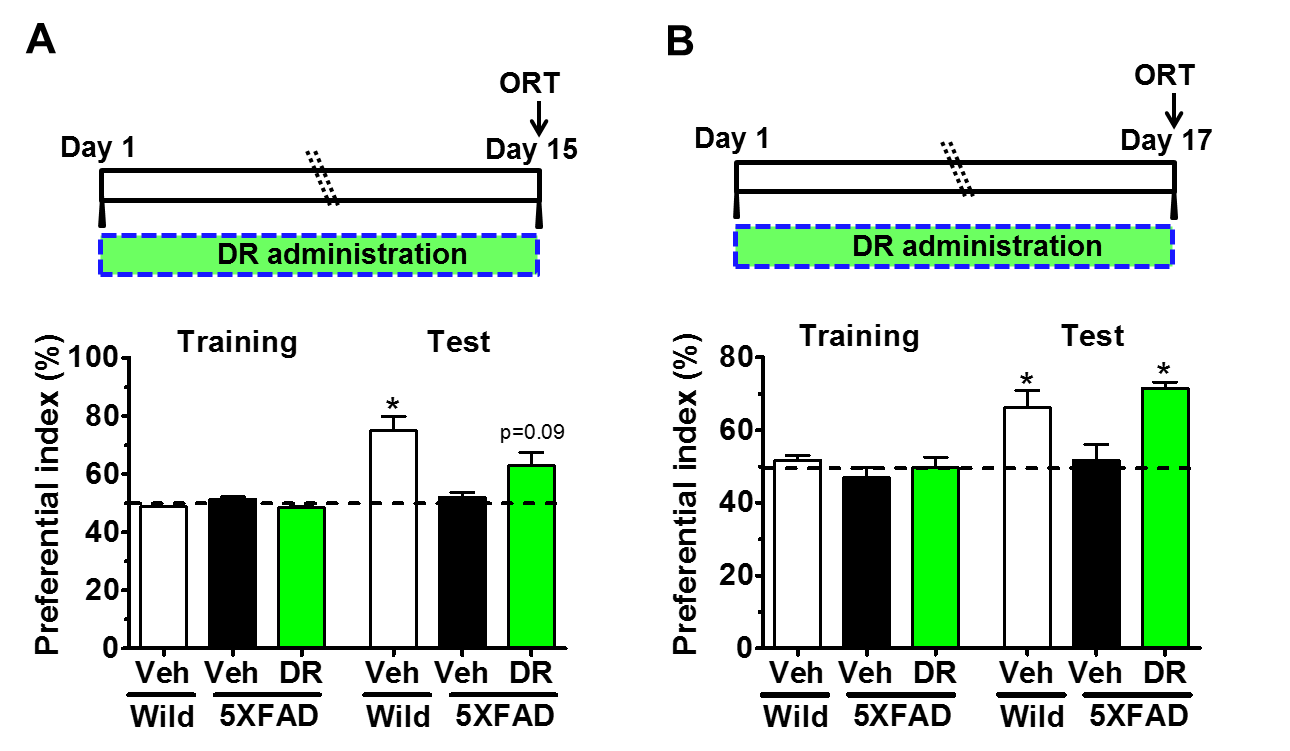
**

**Supplementary Figure 1| DR extract enhances memory function in 5XFAD mice.** **(A)** Six- to fourteen-month-old wild-type mice and 5XFAD mice were used (n = 3). The preference indices were quantified. p = 0.012, drug × time interaction was analysed using repeated measures two-way ANOVA, F(2, 6) = 10.04. *p < 0.05, *post hoc* Bonferroni test. **(B)** Eight-month-old wild-type mice and 5XFAD mice were used (n = 4). The preference indices were quantified. p = 0.135, drug × time interaction was analysed using repeated measures two-way ANOVA, F(2, 7) = 2.70. *p < 0.05, *post hoc* Bonferroni test.


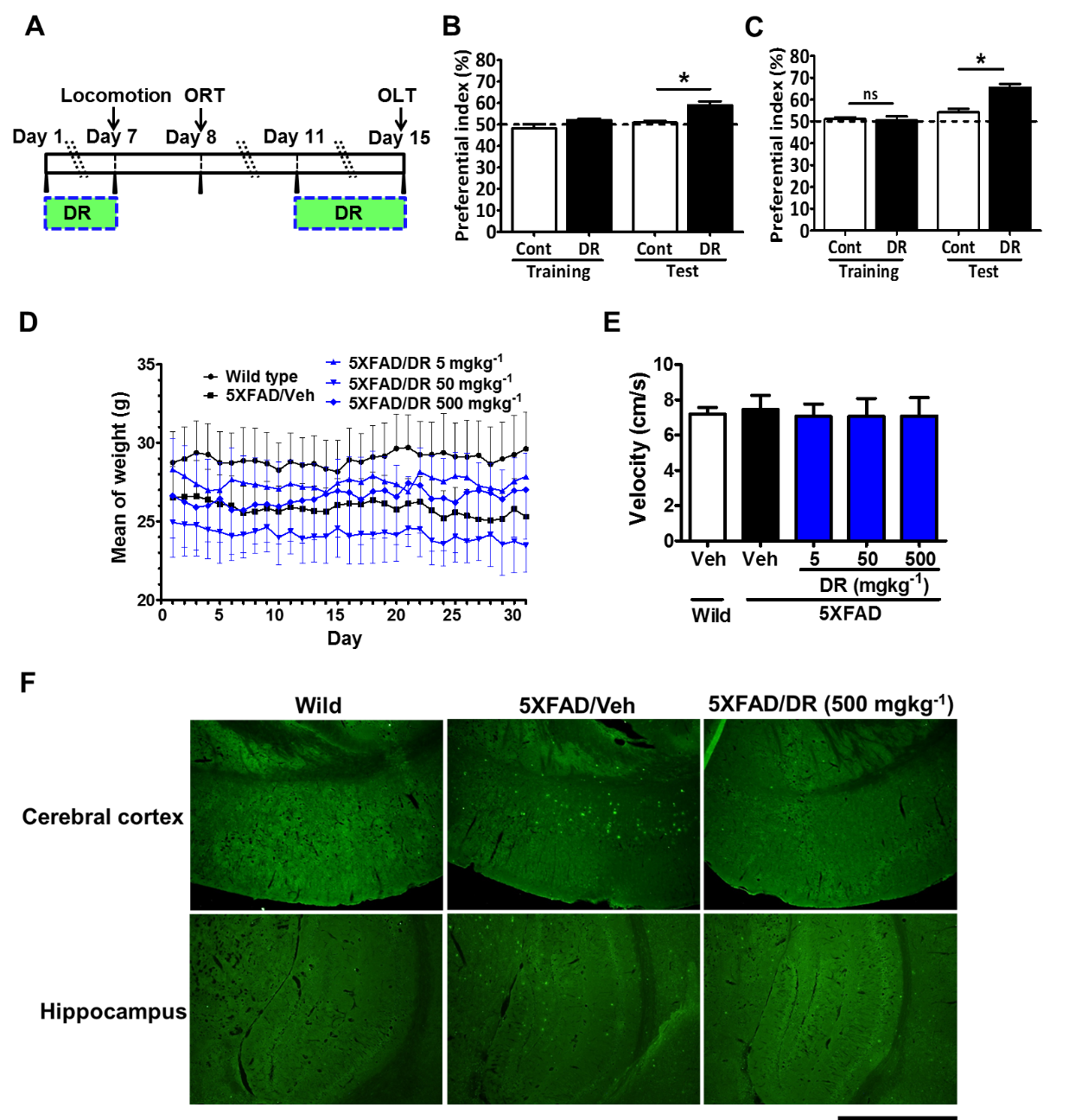


**Supplementary Figure 2| DR extract enhances memory function in normal mice and decreases Aβ plaques in 5XFAD mice.** The effect of DR extract on the memory function in normal mice (a-c). DR (500 mg/kg, p.o.) or vehicle solution was administered for 7 days to mice (males, 6 weeks old). On the day 8 and 15, object recognition and object location test were started, the interval time between training and test was 48 h. The preference indices of the training and test sessions are shown. (**A**) Time course of the experiments. (**B**) Object recognition test. *p* = 0.09, drug × time interaction was analysed using repeated measures Two-way ANOVA, F(1, 8) = 3.58. **p* < 0.05, *post hoc* Bonferroni test. (**C**) Object location test. *p* = 0.005, drug × time interaction was analysed using a repeated measures Two-way ANOVA, F(1, 8) = 14.38. **p* < 0.05, *post hoc* Bonferroni test. The effect of DR extract~~s~~ on 5XFAD mice (d-f). (**D**) The body weight of wild-type mice and 5XFAD mice (Non-repeated two-way ANOVA test, *n* = 3–5 mice). (**E**) The velocity (within 10 min) in the open field test (One-way ANOVA *post hoc* Dunnett’s test, *n* = 3–5 mice). (**F**) Representative images of Aβ_1-40/42_-positive plaques in perirhinal cortex and hippocampus. Scale bar, 1 mm.


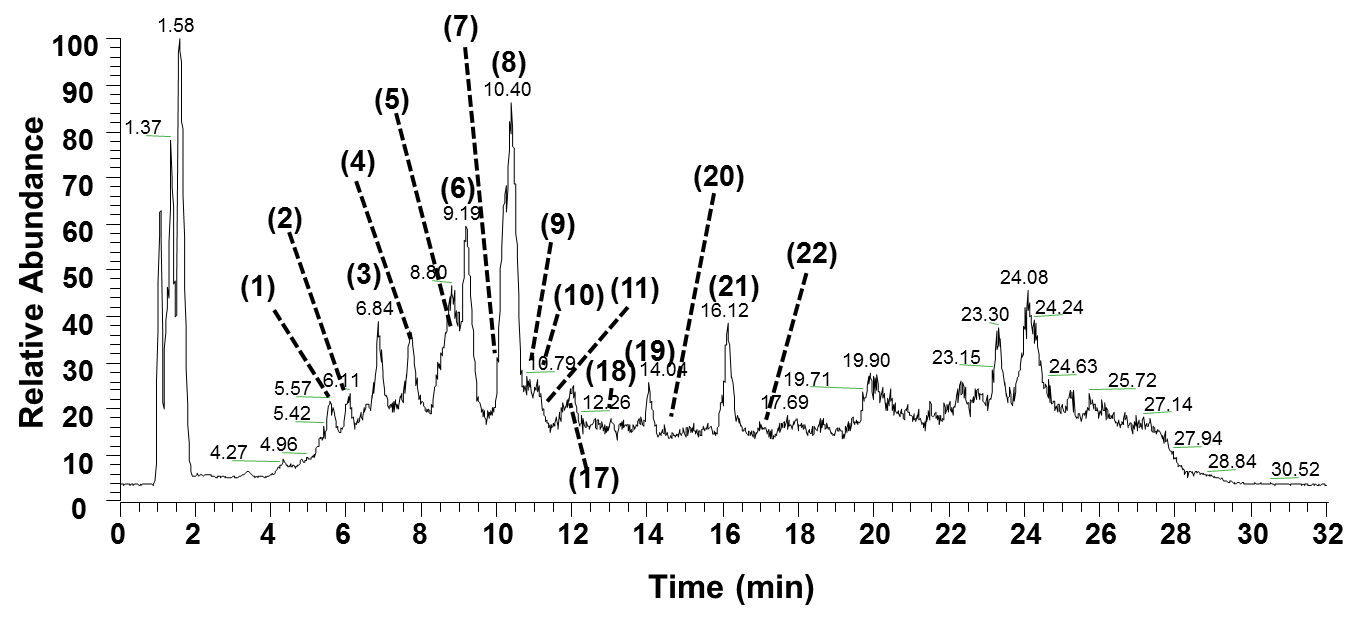


**Supplementary Figure 3| HPLC-FTMS base peak ion chromatogram of the DR extract in the positive ESI mode.** The numbers in each parenthesis correspond to the chemicals listed in supplementary table 1.


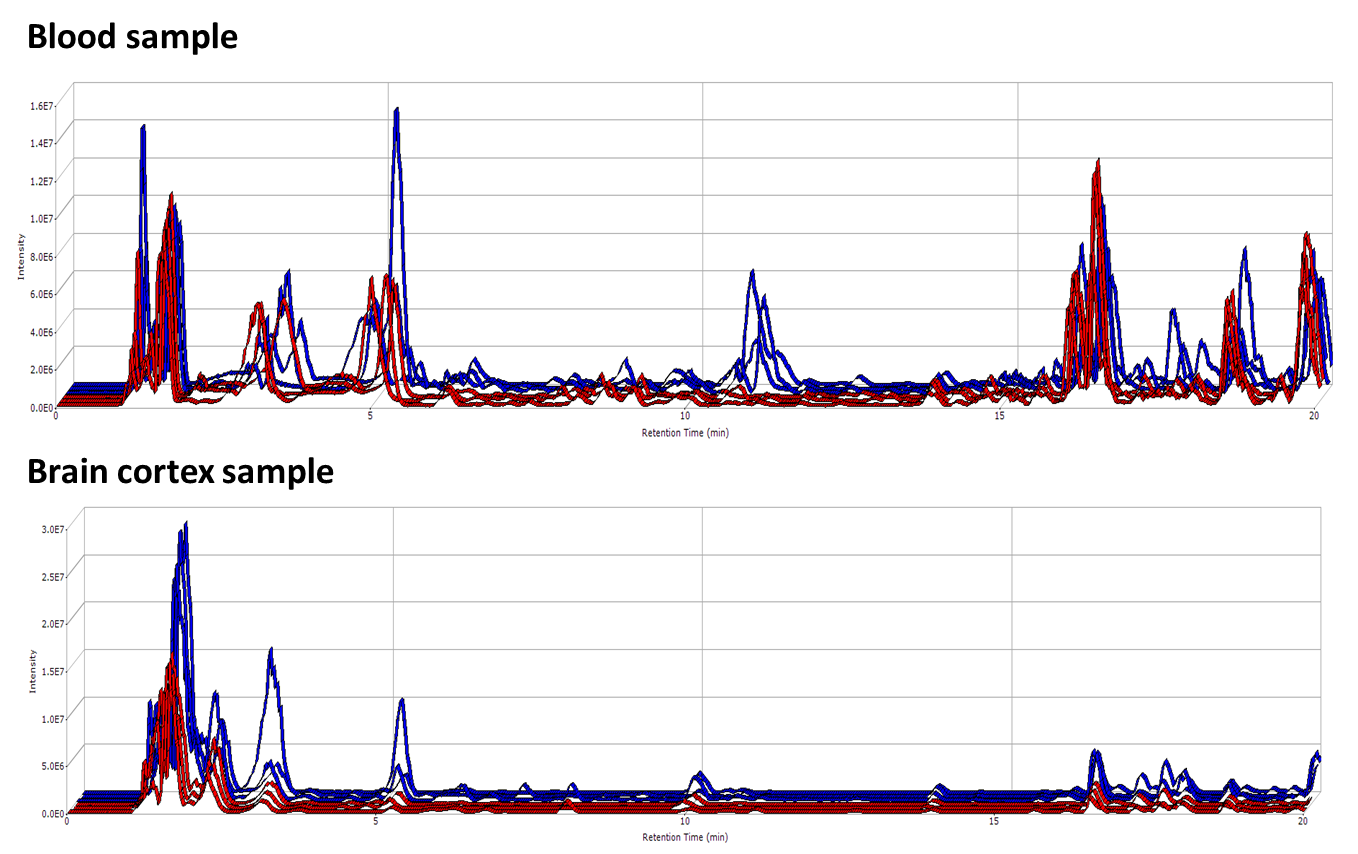


**Supplementary Figure 4| Differential analyses of total ion current (TIC) chromatogram between vehicle treated (red) and DR (13 g kg^-1^) treated (blue) 5XFAD mice biosamples.** DR extract (13 g kg^-1^, p.o.) or vehicle solution (saline) was singly administered to mice (males and females, 12 months old, *n* = 3). Five hours after treatment, mice were sacrificed and the brain cortex was extracted with methanol and applied to LC/MS analyses. The TICs were compared by SIEVE software.


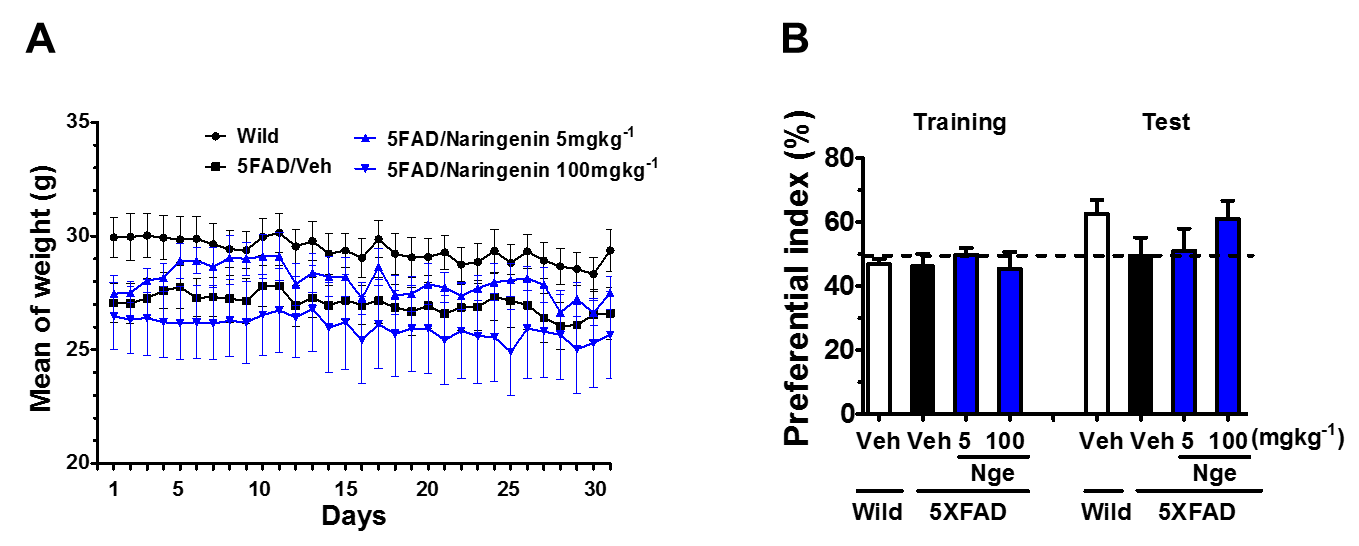


**Supplementary Figure 5| Naringenin ameliorates memory deficits in 5XFAD model mice.** (**A**) The body weight of wild-type mice and 5XFAD mice (Non-repeated two-way ANOVA test, *n* = 4–5 mice). (**B**) Object location test. The preferential indices of the training and test sessions are shown. Drug × time interaction was analysed using repeated measures two-way ANOVA *post hoc* Bonferroni test.


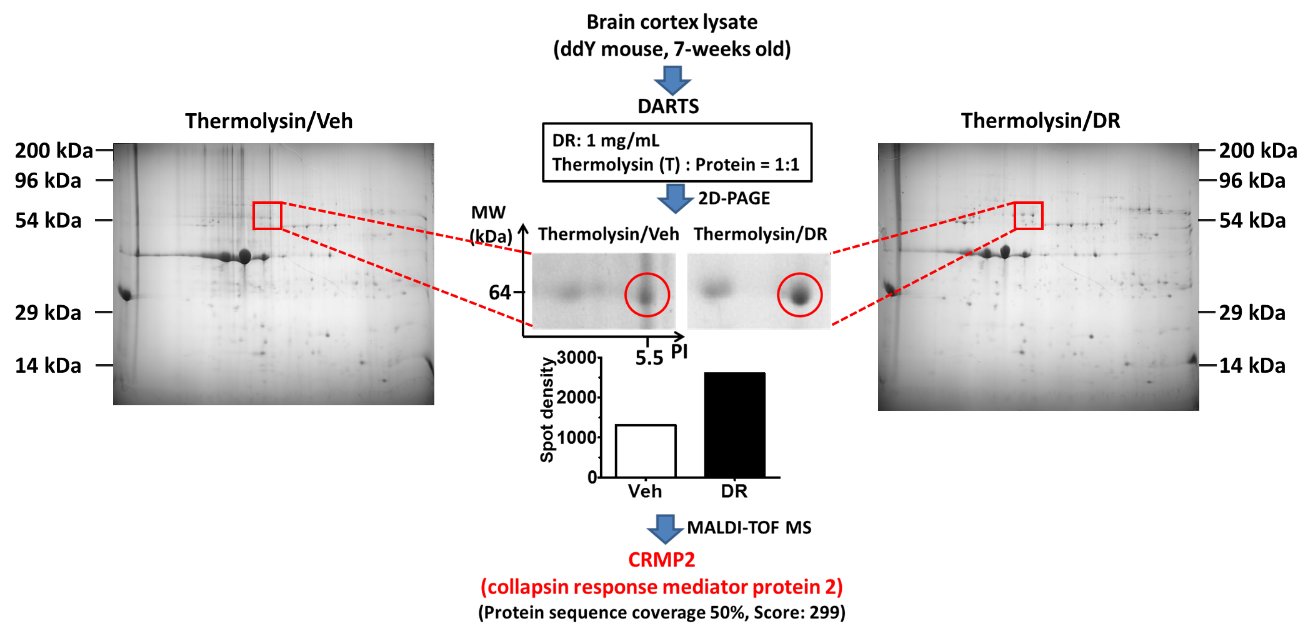


**Supplementary Figure 6| Identification of target proteins of DR extract by DARTS analysis.** Mouse brain cortex was dissected, and the cortex lysate was added to DR (1 mg ml^-1^) or vehicle solution and incubated for 1 h at room temperature. The mixture was proteolysed using thermolysin and subjected to 2D-PAGE analysis. The separated proteins were stained. The spot in the DR-treated lysate was thicker than that in the vehicle-treated lysate, the spots were cut out and prepared for mass spectrometry analysis.


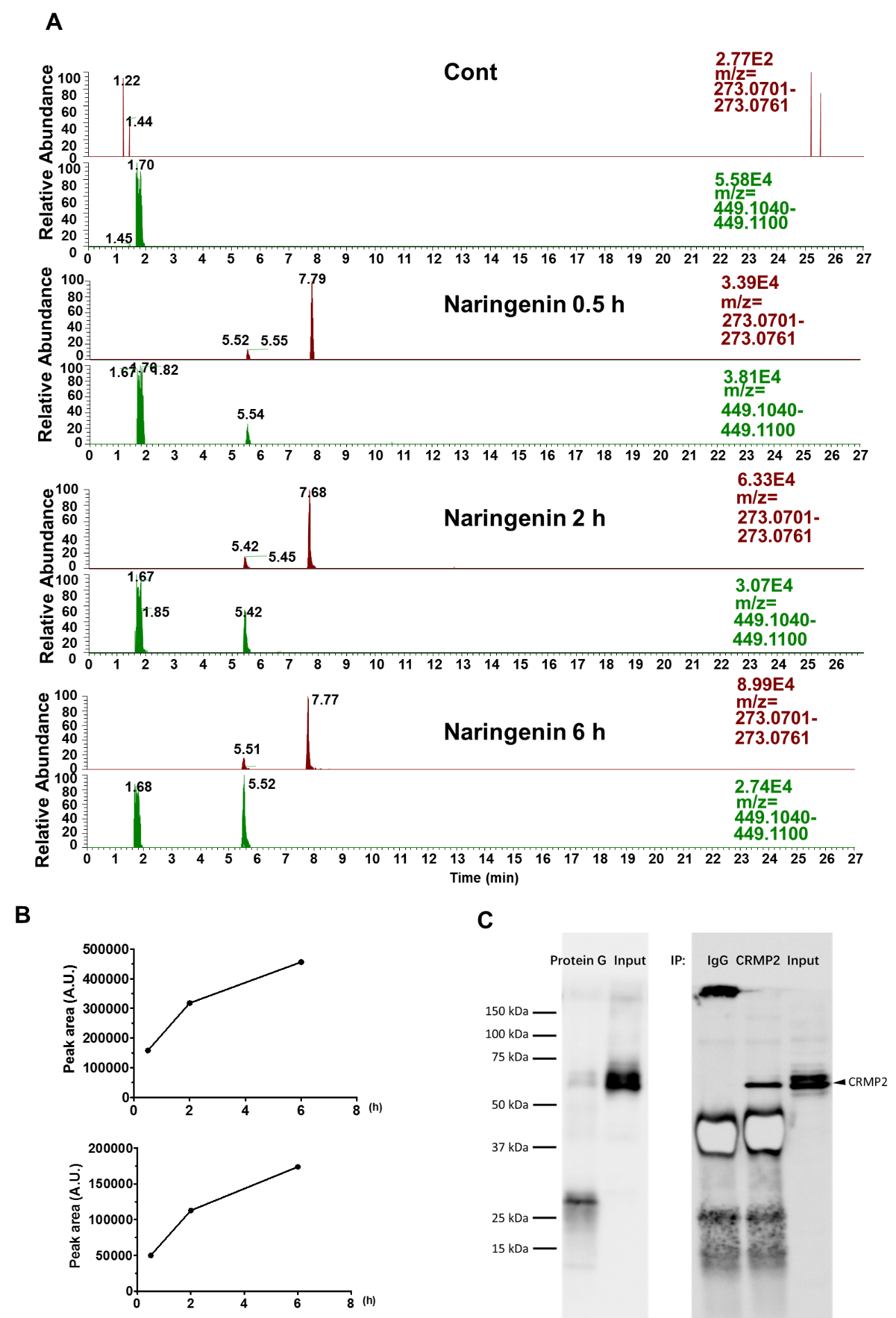


**Supplementary Figure 7| Naringenin could penetrate neuron cell membrane and metabolize to naringenin glucuronide.** (**A**) Naringenin (7.7-7.8 min) and naringenin-7/4’-*O*-glucuronide (5.4-5.5 min) were detected inside of the neurons after incubation with naringenin. The primary mouse cortical neurons (E14) were cultured for three days, and then 100 μM naringenin was treated for 0.5, 2, or 6 h. Neuronal cells were lysed after washing with 1×PBS for three times, 30 μg protein of each was denatured and extracted with methanol, and then was subjected to LC-MS. (**B**) The amount of naringenin (upper panel) or naringenin-7-*O*-glucuronide (lower panel) in the neurons was determined. (**C**) Immunoprecipatation of CRMP2 protein in cultured primary neurons. Primary cultured neurons were maintained for three days, in the left figure, cell lysates (30 µg) were treated with protein G beads (20 µl) for 10 min at 4︒C, protein G beads after boiling were applied to SDS-PAGE, cell lysates (5 μg) was applied as an input. In the right figure, cell lysates were immunoprecipitated by CRMP2 antibody (middle lane) or normal IgG (left lane). Cell lysates (3 μg) was applied as an input (right lane). Western blot was performed by using anti-CRMP2 antibody.


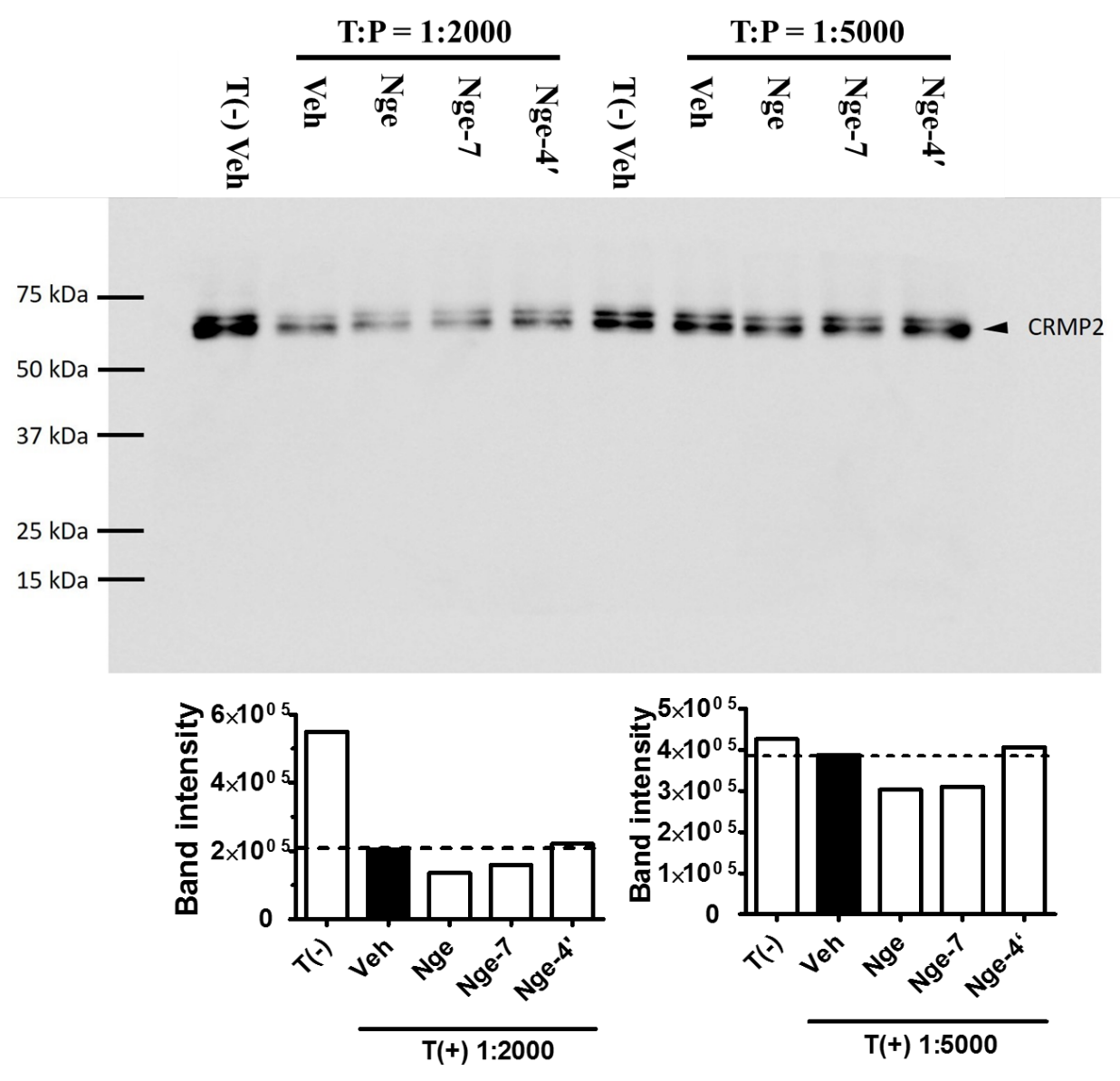


**Supplementary Figure 8| Identification of CRMP2 as a target of naringenin and naringenin-7-*O*-glucuronide by DARTS and western blot analysis.** Mouse cortical neurons (ddY, E14) were cultured for 3 days, and then neurons were lysed with M-PER solution. Naringenin (Nge, 1.67 mM), Naringenin-4’-*O*-glucuronide (Nge-4’, 1.67 mM), Naringenin-7-*O*-glucuronide (Nge-7, 1.67 mM) or vehicle solution was added to the lysates (2.5 μg of each), and the solution was incubated for 1 h at room temperature. The protein (P) mixture was proteolysed using thermolysin (T, 1.25 ng or 0.5 ng) in reaction buffer for 15 min at 37°C. The reaction was stopped by adding 0.5 M EDTA (pH 8.0) to each sample at a 1:10 ratio. The samples were then subjected to SDS-PAGE for western blot analysis.


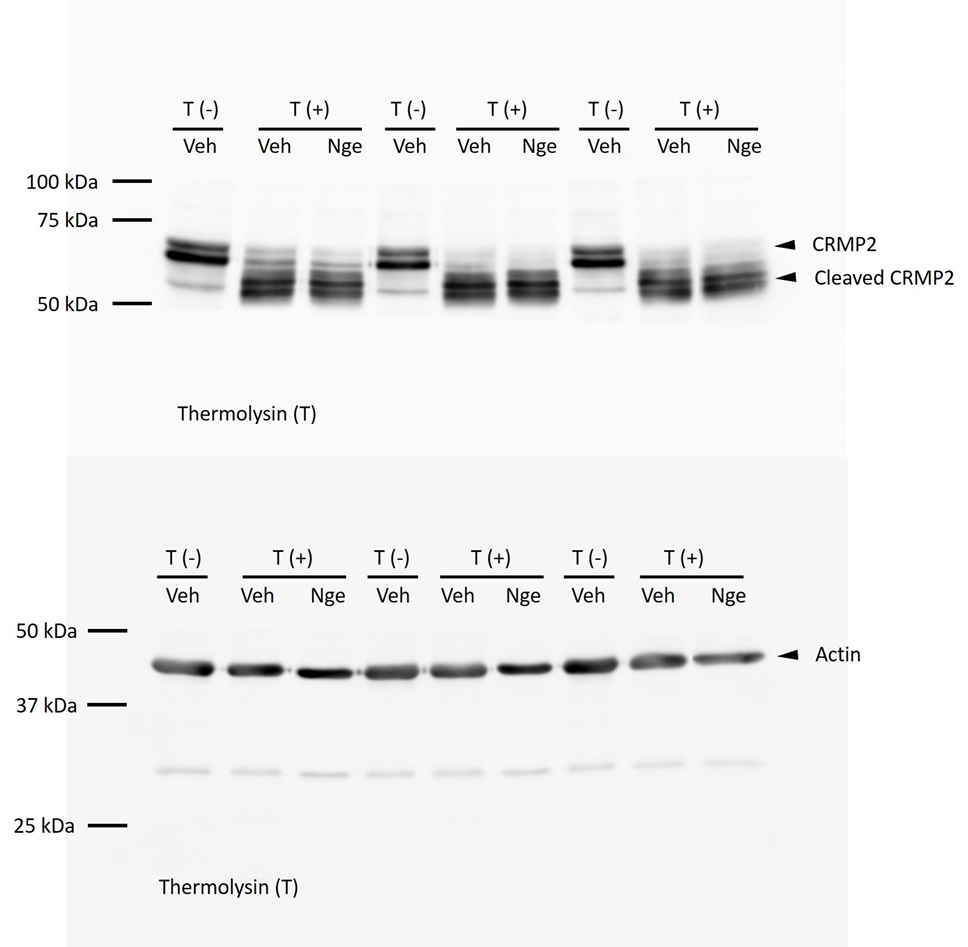


**Supplementary Figure 9| Full length blots of CRMP2 and actin after DARTS and western blot analysis.** Mouse cortical neurons were lysed, naringenin (Nge) or vehicle (Veh) solution was incubated with lysates for 1 h at room temperature. The mixture was proteolysed using thermolysin and electrophoresed for western blot.

**Supplementary Table 1. Compounds identified in DR extract and metabolites that were transferred in plasma and brain following oral administration of DR in 5XFAD mice.**

| Peak No. and Metabolites | RT (min) | MS/MS Fragments | Molecular Formula | [M+H]^+^  (m/z) | Identified Compound | DR | Plasma | Brain |
| --- | --- | --- | --- | --- | --- | --- | --- | --- |
| 1 | 5.59 | 181.0496 | C_15_H_18_O_9_ | 343.1025 | Caffeic acid-4-*O*-glucoside | + | +(0.5h) | - |
| 2 | 6.06 | 579.1496 (427.0313, 409.0839, 291.1580, 289.0319, 247.1223), 289.0708(271.0996, 163.0733, 127.1166) | C_30_H_26_O_12_ | 579.1496 | Proanthocyanidin B | + | - | - |
| 3 | 6.86 | 341.0869, 179.0339 | C_21_H_26_O_13_ | 487.1447 | 5,7-dihydroxychromone-7-neohesperidoside | + | +(0.5, 5h) | - |
| 4 | 7.70 | 449.1080, 287.0552 (269.1473, 241.1353, 213.0526, 165.1339, 153.0798) | C_27_H_30_O_15_ | 595.1658 | Kaemperol-3-*O*-rutinoside | + | - | - |
| 5 | 8.35 | 287.0551 (269.0333, 259.0746, 241.1809, 231.0298, 153.1035) | C_27_H_30_O_15_ | 595.1657 | Kaemperol-7- neohesperidoside | + | - | - |
| 6 | 9.17 | 451.1239, 435.1290, 289.0710(163.0673, 153.1316), 153.0183 | C_27_H_32_O_15_ | 597.1818 | Neoeriocitrin | + | +(0.5, 5h) | - |
| 7 | 9.40 | 419.0974, 287.0551 (153.0846) | C_27_H_30_O_15_ | 595.1657 | Luteolin-7-neohesperidoside | + | - | - |
| 8 | 10.39 | 435.1287, 419.1339, 273.0757(153.0337, 147.0683), 153.0182 | C_27_H_32_O_14_ | 581.1866 | Naringin | + | +(0.5, 5h) | - |
| 9 | 10.66 | 287.0550 (269.1468, 258.0283, 241.1390, 231.1378, 213.1349, 165.0254, 153.0266) | C_21_H_20_O_11_ | 449.1080 | Kaemperol-3-*O*-glucoside | + | - | - |
| 10 | 10.81 | 273.0761(153.0331, 147.0550) | C_21_H_22_O_10_ | 435.1289 | Naringenin-7-*O*-glucoside | + | +(0.5h) | - |
| 11 | 11.08 |  | C_9_H_6_O_4_ | 179.0339 | 5,7-dihydroxychromone | + | +(0.5, 5h) | - |
| M1 | 5.47 | 179.0335 | C_15_H_14_O_10_ | 355.0654 | 5,7-dihydroxychromone-glucuronide | - | +(0.5, 5h) | - |
| M2 | 9.55 | 179.0334 | C_9_H_6_O_7_S | 258.9900 | 5,7-dihydroxychromone-sulfate | - | +(0.5, 5h) | - |
| M3 | 9.55, 10.40, 10.92 | 289.0699 | C_21_H_20_O_12_ | 465.1010 | Eriodictyol-glucuronide | - | +(0.5, 5h) | - |
| M4 | 10.65 | 273.0750 | C_21_H_20_O_11_ | 449.1063 | Naringenin-7-*O*-glucuronide | - | +(0.5, 5h) | +(5h) |
| M5 | 11.18 | 273.0750 | C_21_H_20_O_11_ | 449.1063 | Naringenin-4’-*O*-glucuronide | - | +(0.5, 5h) | +(5h) |
| M6 | 12.36 | 273.0750 | C_21_H_20_O_11_ | 449.1063 | Naringenin-chalcone-glucuronide | - | +(0.5, 5h) | - |
| 17 | 12.01 | 287.0551(269.0756, 258.0622, 241.0635, 231.0604, 213.0176,165.0438, 153.0968), 179.0339 | C_21_H_19_O_10_ | 433.1129 | Kaemperol-3-*O*-rhamnoside | + | - | - |
| 18 | 13.03 | 287.0551 (269.1929, 258.1298, 241.1409, 213.0502, 165.0406, 153.0382) | C_20_H_18_O_10_ | 419.0974 | Kaempferol 3-*O*-arabinoside | + | - | - |
| 19 | 14.04 | 163.0673, 153.1316 | C_15_H_12_O_6_ | 289.0710 | Eriocictyol | + | - | - |
| 20 | 14.40 | 269.1717, 153.0713 | C_15_H_10_O_6_ | 287.0552 | Luteolin | + | - | - |
| 21 (M7) | 16.15 | 153.0658, 147.1258 | C_15_H_12_O_5_ | 273.0760 | Naringenin | + | +(5h) | +(5h) |
| 22 | 16.70 | 287.0760, 269.1448, 241.1417, 213.2002, 165.0957, 153.0219 | C_15_H_10_O_6_ | 287.0551 | Kaempferol | + | - | - |
